# RTP4, a Biomarker Associated with Diagnosing Pulmonary Tuberculosis and Pan-Cancer Analysis

**DOI:** 10.1155/2023/2318473

**Published:** 2023-04-26

**Authors:** Hao Li, Qin Zhou, ZhiXiang Ding, QingHai Wang

**Affiliations:** ^1^Department of Infectious Diseases, The First People's Hospital of Changde City, Changde, China; ^2^Intensive Care Unit, The First People's Hospital of Changde City, Changde, China

## Abstract

**Background:**

Pulmonary tuberculosis (PTB) is a global epidemic of infectious disease; the purpose of our study was to explore new potential biomarkers for the diagnosis of pulmonary tuberculosis and to use the biomarkers for further pan-cancer analysis.

**Methods:**

Four microarray gene expression sets were downloaded from the GEO public databases and conducted for further analysis. Healthy control (HC) samples and samples of pulmonary tuberculosis (PTB) were calculated with enrichment scores in folate biosynthesis pathways. The scores acted as a new phenotype combined with clinical information (control or PTB) for subsequent analysis. Weight gene coexpression network analysis (WGCNA) was used to seek the modules mostly related to PTB and folate biosynthesis in training sets. Twenty-nine coexistence genes were screened by intersecting the genes in the green-yellow module of GSE28623 and the brown module of GSE83456. We used the protein-protein interaction network analysis to narrow the gene range to search for hub genes. Then, we downloaded the unified and standardized pan-cancer data set from the UCSC database for correlations between biomarkers and prognosis and tumor stage differences.

**Results:**

Eventually, RTP4 was selected as a biomarker. To verify the reliability of this biomarker, an area under the ROC (AUC) was calculated in gene sets (GSE28623, GSE83456, and GSE34608). Lastly, to explore the difference in RTP4 expression before and after antituberculosis treatment, the GSE31348 gene set was enrolled to compare the expressions in weeks 0 and 26. The results showed significant differences between these two time points (*p* < 0.001). RTP4 was significantly upregulated in the pulmonary tuberculosis group compared to the healthy control group in three gene sets and downregulated after antituberculosis therapy in one gene set. These results suggest that RTP4 can be used as a potential biomarker in diagnosing tuberculosis. The results of pan-cancer analysis showed that high expression of RTP4 in 4 tumor types was positively correlated with poor prognosis and high expression of RTP4 in 6 tumor types was negatively correlated with poor prognosis. We found significant differences in the expression of the RTP4 gene at different stages in 5 types of tumors.

**Conclusion:**

RTP4 might be a new potential biomarker for diagnosing pulmonary tuberculosis.

## 1. Introduction

Mycobacterium tuberculosis (MTB) is one of the significant causes of tuberculosis. According to the World Health Organization, about 5.8 million people worldwide were infected with TB in 2020 [1]. Mycobacterium tuberculosis infection has become a global public problem, especially in developing countries [2].

MTB can escape immune surveillance and kill by inhibiting oxidative stress, autophagy, and apoptosis of cells. It also can affect antigen presentation of antigen-presenting cells (APC) by inhibiting the synthesis of histocompatibility complex molecules [3]. There are many clinical trials used to test for diagnosing TB, such as tuberculin skin test (TST), T-SPOT, smear microscope, the culture of Mycobacterium tuberculosis, and chest X-ray [4, 5]. But as a slow-growing intracellular parasitic bacteria [6], the above clinical test methods have certain false negative and hysteresis.

To search for sensitive indicators in Mycobacterium tuberculosis infection, we use bioinformatics methods to compare and analyze the spectrum of gene expression spectrum of pulmonary tuberculosis and healthy people, hoping to find genes that play a significant role in the pathogenesis of PTB as potential biomarkers for diagnosing pulmonary tuberculosis and further explore the relationship between biomarkers and tumor prognosis and stage through pan-cancer analysis.

## 2. Materials and Methods

### 2.1. Data Acquisition and Processing

We downloaded the GSE28623 (GPL4133, Agilent-014850 Whole Human Genome Microarray), GSE83456 (GPL10558, Illumina HumanHT-12 V4.0 expression beadchip), GSE34608 (GPL6480, Agilent-014850 Whole Human Genome U133 Microarray), and GSE31348 (GPL570, Affymetrix Human Genome U133 Plus) from Gene Expression Omnibus (GEO, https://www.ncbi.nlm.nih.gov/geo/) by “GEOquery” R-package and extracted each expression profile information and Clinical phenotype of these gene sets. There were 46 pulmonary tuberculosis samples and 37 healthy control samples in GSE28623 and 45 pulmonary tuberculosis samples and 61 healthy control samples in GSE83456. Finally, 189 blood samples were enrolled as training sets. There were 8 pulmonary tuberculosis samples and 16 healthy control samples in GSE34608 and 135 pulmonary tuberculosis samples in GSE31348. In the end, 159 blood samples were enrolled as validation sets. After that, we downloaded the unified and standardized pan-cancer data set from the UCSC (https://xenabrowser.net/) database, TCGA Pan-cancer (PANCAN, *N* = 10535, *G* = 60499), extracted expression data of the RTP4 gene (ENSG00000136514) in each sample, and carried out a log2 (*x* + 0.001) transformation, and finally, we also eliminated cancer types with fewer than 3 samples in a single cancer type and finally obtained the expression data of 26 cancer types.

### 2.2. KEGG and Gene Set Variation Analysis

The KEGG was a set of databases that included information about biological mechanisms, cellular processes, chemical substances, and diseases [7]. Gene set variation analysis (GSVA) was applied to explore different activity variations of the KEGG pathway (c2.cp.kegg v7.5.1, http://www.gsea-msigdb.org/) in GSE28623 and GSE83456 by using “GSVA” R package [8]. “limma” R package was used to determine the significant differences in GSVA enrichment between the healthy control (HC) group and pulmonary tuberculosis (PTB) group. After setting the threshold value (PTB versus HC, log2FC > 0.25, adjust *p* value < 0.05) and taking the intersection of the training set (GSE28623 and GSE83456), we selected the folate biosynthesis pathway enrichment scores for further analysis with clinical phenotype.

### 2.3. Identification of Significant Modules and Weight Gene Coexpression Network Analysis

Data selected in training sets were processed using the “WGCNA” R package [9]. After obtaining the gene expression matrix of GSE28623 and GSE83456 gene sets, we chose the genes that variance in the top quartile and used the hierarchical agglomerative clustering (average link) to distinguish the outliers of each gene set. Then, we set each threshold to construct a scale-free network. After setting an approximate scale-free topology fit index above 0.85, we built an adjacency matrix and constructed the topological overlap matrix for searching the coexpression modules, which is a collection of genes with high topological overlap similarity and containing at least 30 genes. Genes in the same module usually have a higher degree of coexpression. The module eigengene (ME) represents the first principal component of modules and reflects the expression pattern of modules in each sample. After merging modules whose similarities were higher than 75%, 8 modules in GSE28623 and 13 modules in GSE83456 were identified according to the average hierarchical clustering and dynamic tree clipping algorithm. The green-yellow module of GSE28623 and brown module of GSE83456 were most relevant to the clinical phenotype and folate biosynthesis pathway enrichment.

### 2.4. Hub Gene Identification and PPI Network

The coexisting genes in both modules were considered essential and uploaded to the Search Tool for the Retrieval of Interacting Genes (STRING) online database (http://string-db.org; version 11.5). A confidence score > 0.70 was set as significant. After obtaining the protein-protein interaction (PPI) network data, we used the Cytoscape (version 3.9.1) to visualize the results. In order to verify the effectiveness of the hub genes, “pROC” and “ggplot2” were used to calculate and plot the area under curve (AUC) [10, 11].

### 2.5. Pan-Cancer Analysis

We used R software to calculate the expression difference between normal samples and tumor samples in each tumor to analyze the significance of the difference. The “Coxph” function of “survival” R package (version 3.4.0) was used to establish a Cox proportional hazard regression model to analyze the relationship between gene expression and prognosis in each tumor. After that, we calculated the expression difference of genes in different clinical stage samples in each tumor to explore the correlations between them.

### 2.6. Statistical Analysis

This study's statistical analyses were carried out by R (version 4.1.0). Linear fitting and empirical Bayes, implemented in the “limma” R package, were used to test the difference between the HC and PTB groups [12]. Weight gene coexpression network analysis was used to allocate genes with similar expression patterns into different modules. The Pearson correlation analysis was applied to find correlations between the selected modules and clinical phenotype. The receiver operating characteristic (ROC) curve was used to evaluate the reliability of candidate biomarkers. It was considered statistically significant if the area under curve (AUC) was greater than 0.70. Student's *t*-test was used to estimate the different expressions of RTP4 before and after antituberculosis therapy. All *p* < 0.05 (bilateral) was considered statistically significant. The logarithmic ranking test was to obtain the tumor prognostic significance. The unpaired Student's*t*-test was used for analyzing difference between each type of tumor groups. The unpaired Wilcoxon rank sum and signed rank tests were used to explore the significance of the difference between normal and tumor samples in each tumor type.

## 3. Results

### 3.1. Folate Biosynthesis Pathway Gene Set Scores and WGCNA

**Figure 1:**
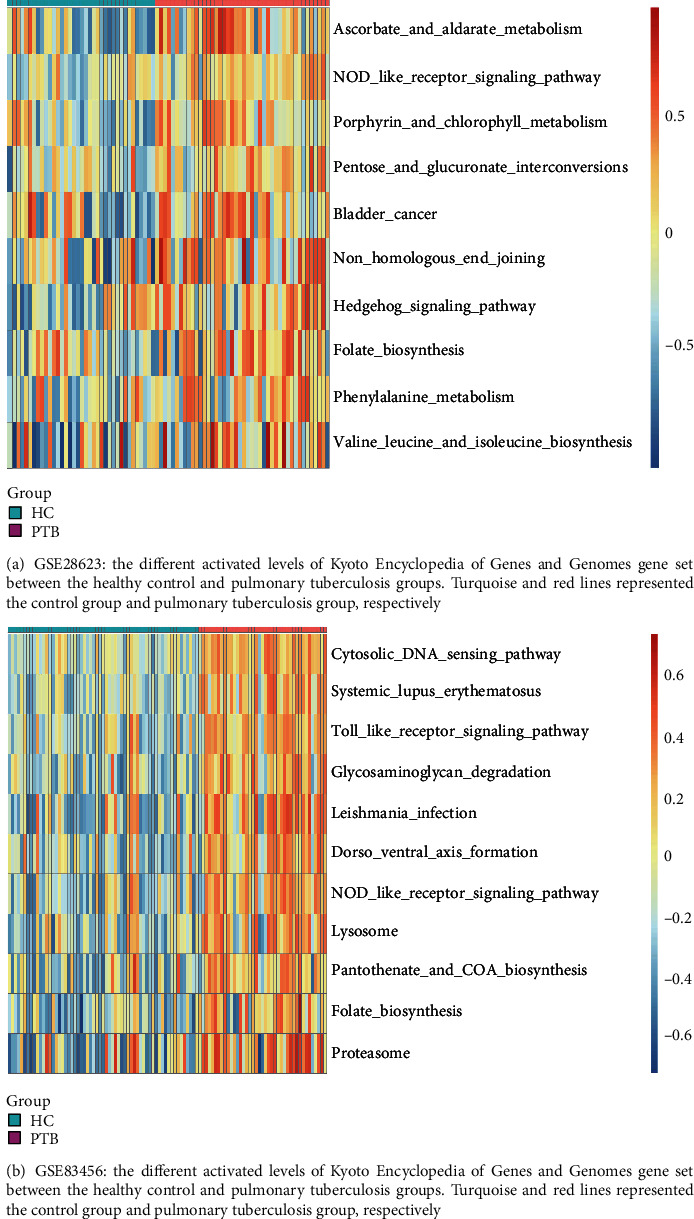
Enrichment of differential KEGG pathway gene sets of training gene sets.

**Figure 2:**
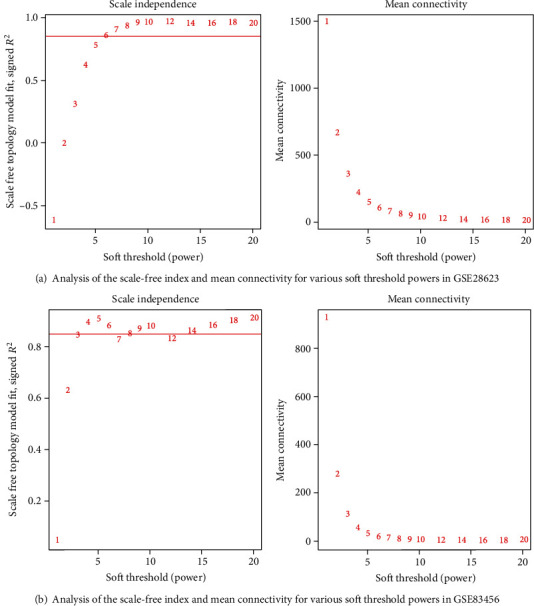
Determination of soft threshold power in the WGCNA.

**Figure 3:**
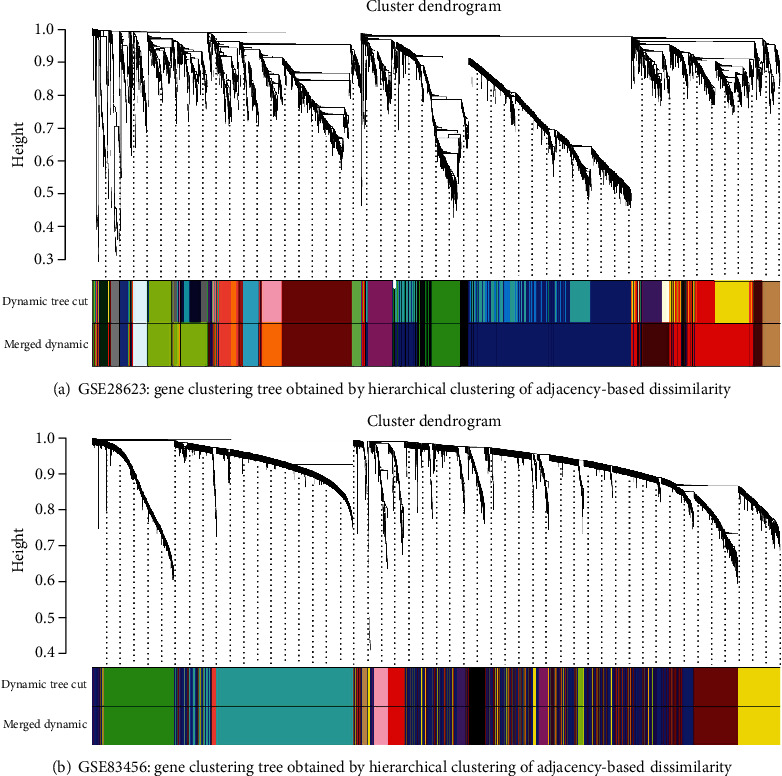
Dendrogram of all differentially expressed genes clustered based on the measurement of dissimilarity (1-TOM). The color band shows the results obtained from the automatic single-block analysis. Each color band of dynamic tree cut represents a cluster of a collection of genes with high topological overlap similarity and has minimal genes of 30. Merged dynamic represents the merged modules of dynamic tree cut, in which the dissimilarity degree is below 75%.

**Figure 4:**
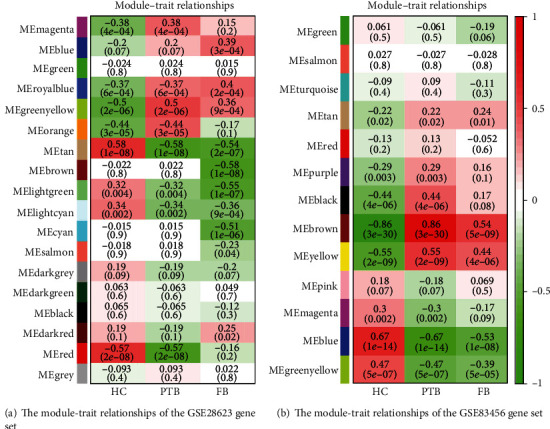
Each row corresponds to the module eigengene column to a clinical trait. Each cell contains the corresponding correlation and *p* value, red for positive and green for negative correlations.

The gene expression of GSE28623 (19172 genes) and GSE83456 (20937 genes) was obtained after data preprocessing. After excluding latent tuberculosis, extrapulmonary tuberculosis, and sarcoid samples, we had 83 samples left in GSE28263 and 106 samples in GSE83456. We used an agglomerative hierarchical clustering algorithm to exclude 2 PTB samples of GSE28623 and 4 PTB samples of GSE83456 because there were outliers. Subsequently, the top quartile variance genes were selected (GSE28623, 4982 genes; GSE83456, 5234 genes). Through gene set variation analysis, we found that the KEGG pathways activated in GSE28623 (PTB group versus HC group) were mainly associated with ascorbate and aldarate metabolism, NOD-like receptor signaling, porphyrin and chlorophyll metabolism, pentose and glucuronate interconversions, bladder cancer, nonhomologous and joining, hedgehog signaling, folate biosynthesis, phenylalanine metabolism, valine leucine, and isoleucine biosynthesis. However, the main KEGG pathways activated in GSE83456 (PTB group versus HC group) were cytosolic DNA sensing, systemic lupus erythematosus, toll-like receptor signaling, glycosaminoglycan degradation, leishmania infection, dorso-ventral axis formation, NOD-like receptor signaling, lysosome, pantothenate and CoA biosynthesis, folate biosynthesis, and proteasome. We observed the same activation trend both in GSE28623 (log2FC = 0.286, adj.*p* < 0.001) and GSE83456 (log2FC = 0.325, adj.*p* < 0.001) of folate biosynthesis pathway. The differential KEGG pathway gene set enrichment results were depicted by a heat map (Figure 1). We set the threshold power at 6 (GSE28623, *R*^2^ = 0.850) and 4 (GSE83456, *R*^2^ = 0.895) respectively based on an approximate scale-free topology fit index of above 0.85 for each gene set. This network conforms to the power-law distribution closer to the real biological network state (Figures 2(a) and 2(b)). Gene dendrograms and module colors of the training sets are shown in Figure 3. After obtaining the different modules, we screened out that the most associated with pulmonary tuberculosis. After that, we selected the most relevant folate biosynthesis pathway of them. By carefully comparing, we found that the green-yellow module in GSE28623 and the brown module in GSE83456 were highly related to pulmonary tuberculosis and the folate biosynthesis pathway. Finally, we selected these two modules for further analysis (Figure 4).

### 3.2. PPI Network Analysis and Hub Gene Identification

**Figure 5:**
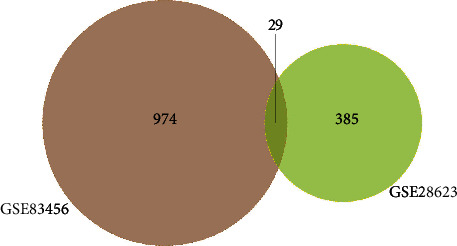
Coexisting genes exist in yellow-green modules (GSE28623) and brown modules (GSE83456). The intersection of the two modules.

**Figure 6:**
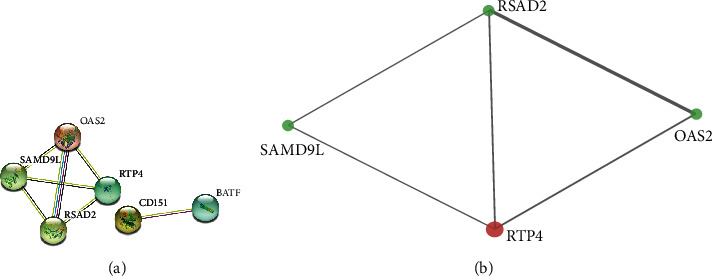
PPI network. (a) The interaction between 6 intersections coexisted; only 4 genes had the interaction. (b) The interaction between hub genes.

In the intersection of the two modules, we obtained 29 coexisting genes, which were illustrated by the Veen map (Figure 5). After using the STRING online database, eight nodes and sixteen edges were observed. Six genes (OAS2, SAMD9L, RSAD2, RTP4, CD151, and BATF) were filtered in the PPI network complex based on the setting confidence score (Figure 6).

**Figure 7:**
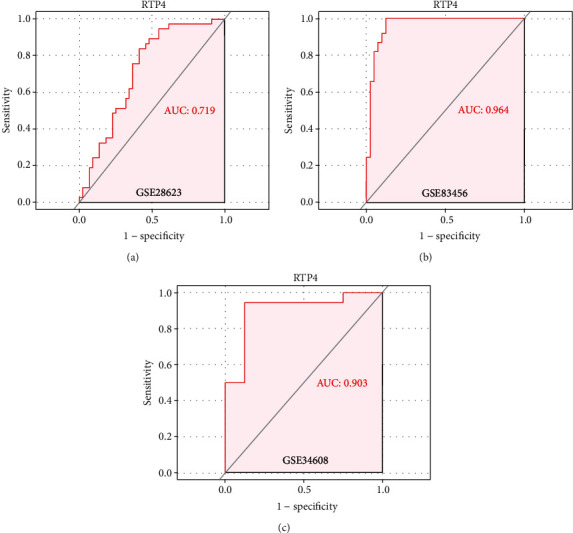
The different AUCs of RTP4 expression. AUC plot of RTP4 expression in GSE28623, GSE83456, and GSE34608, respectively.

**Figure 8:**
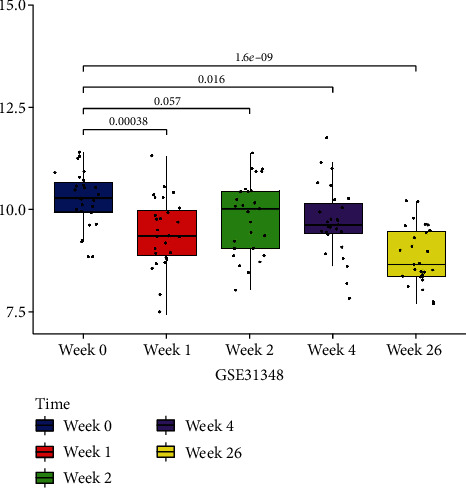
Diversity expression of RTP4 before and after antituberculosis therapy (five time points). RTP4 expression of different time points (week 0, week 1, week 2, week 4, and week 26) in GSE31348.

**Figure 9:**
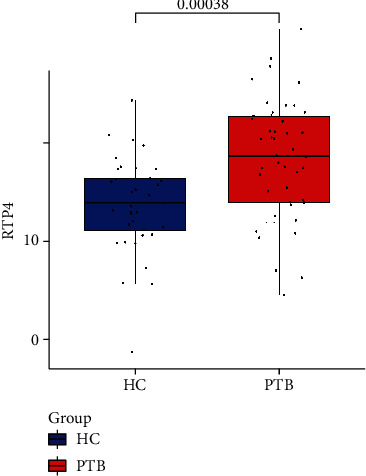
The different expressions of RTP4 between the HC group and PTB group in GSE28623.

**Figure 10:**
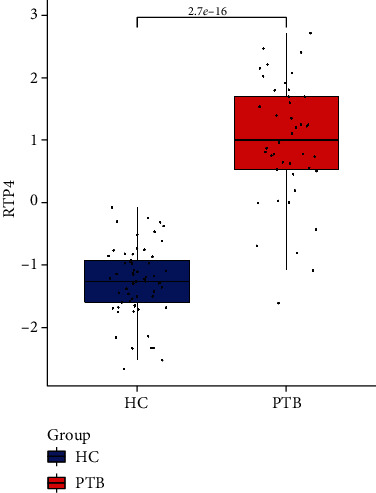
The different expressions of RTP4 between the HC group and PTB group in GSE83456.

We then screen one (RTP4) of them according to the area under curve both in training sets (GSE28623, AUC = 0.719; GSE83456, AUC = 0.964, Figures 7(a) and 7(b)) and the validation cohort (GSE34608, AUC = 0.903Figure 7(c)) as the hub gene. To make the outcome persuasive, we chose the gene set (GSE31384) to validate diversity expression before and after antituberculosis therapy and compare the differential expression of RTP4 between the HC and PTB groups in GSE28623 and GSE83456. As shown in Figure 8, the expression of RTP4 was significantly downregulated after 26 weeks of antituberculosis treatment. We also found that there were significantly upregulated in the PTB group (Figures 9 and 10).

### 3.3. Pan-Cancer Analysis

**Figure 11:**
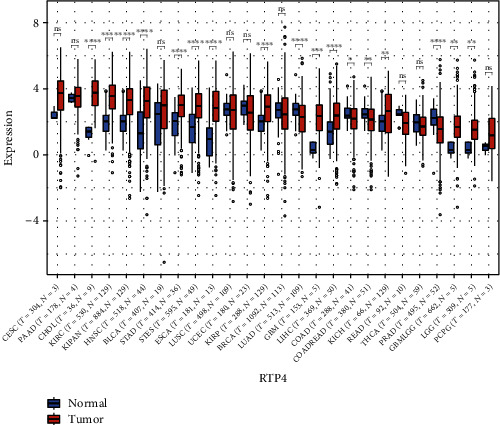
The different expressions of RTP4 in 26 types of tumors between normal samples and tumor samples. ^∗^0.05, ^∗∗^0.01, ^∗∗∗^0.001, and ^∗∗∗∗^0.0001. Red: tumor group. Blue: normal group.

In the expression difference between normal samples and tumor samples in each tumor, we found that RTP4 was significantly upregulated in 13 types of tumor tissues such as GBM (tumor: 2.22 ± 1.14, normal: 0.3 ± 0.44, *p* = 5.4*e* − 4), GBMLGG (tumor: 1.70 ± 1.09, normal: 0.35 ± 0.44, *p* = 3.2*e* − 3), LGG (tumor: 1.54 ± 1.02, normal: 0.35 ± 0.44, *p* = 5.5*e* − 3), ESCA (tumor: 2.78 ± 1.49, normal: 1.00 ± 1.39, *p* = 1.5e − 4), STES (tumor: 2.87 ± 1.24, normal: 1.63 ± 1.20, *p* = 1.7*e* − 10), KIRP (tumor: 2.78 ± 1.34, normal: 2.00 ± 0.76, *p* = 4.2*e* − 13), KIPAN (tumor: 3.15 ± 1.27, normal: 2.00 ± 0.76, *p* = 7.1*e* − 28), STAD (tumor: 2.91 ± 1.12, normal: 1.86 ± 1.06, *p* = 1.1*e* − 7), HNSC (tumor: 3.07 ± 1.50, normal: 1.34 ± 1.51, *p* = 6.5*e* − 11), KIRC (tumor: 3.44 ± 1.10, normal: 2.00 ± 0.76, *p* = 6.7*e* − 37), LIHC (tumor: 2.29 ± 1.29, normal: 1.50 ± 1.19, *p* = 1.3*e* − 6), KICH (tumor: 2.47 ± 1.48, normal: 2.00 ± 0.76, *p* = 2.8*e* − 3), and CHOL (tumor: 3.66 ± 1.28, normal: 1.25 ± 0.56, p = 6.0*e* − 7) and significantly downregulated in 4 types of tumor tissues such as LUAD (tumor: 2.19 ± 1.22, normal: 2.71 ± 0.56, *p* = 1.9*e* − 6), COAD (tumor: 2.08 ± 1.13, normal: 2.51 ± 0.57, *p* = 0.02), COADREAD (tumor: 2.02 ± 1.10, normal: 2.50 ± 0.55, *p* = 2.1*e* − 3), and PRAD (tumor: 1.50 ± 1.27, normal: 2.22 ± 0.75, *p* = 1.7*e* − 6) (Figure 11) [13].

**Figure 12:**
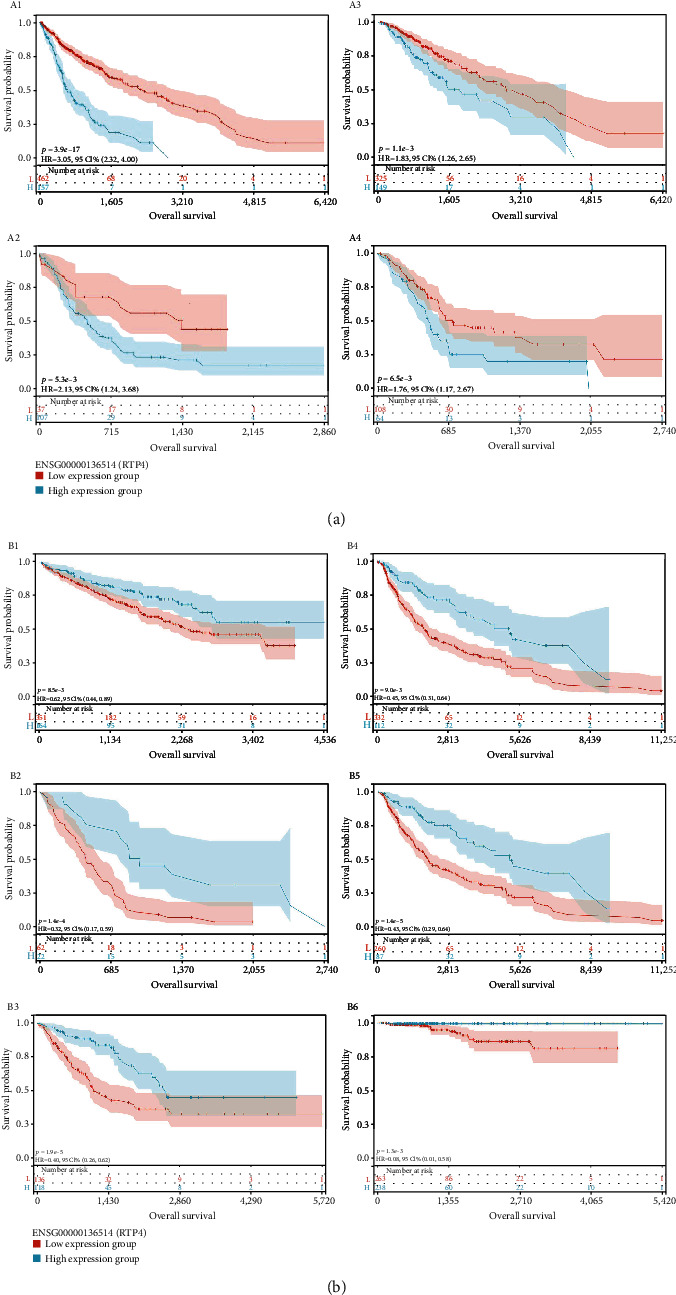
(A1–A4) The survival probability of the RTP4 low expression group and high expression group in GBMLGG, LAMA, LGG, and PAAD. (B1–B6) The survival probability of RTP4 low expression group and high expression group in KIRC, MESO, SARC, SKCM. Red: low expression group. Blue: high expression group.

**Figure 13:**
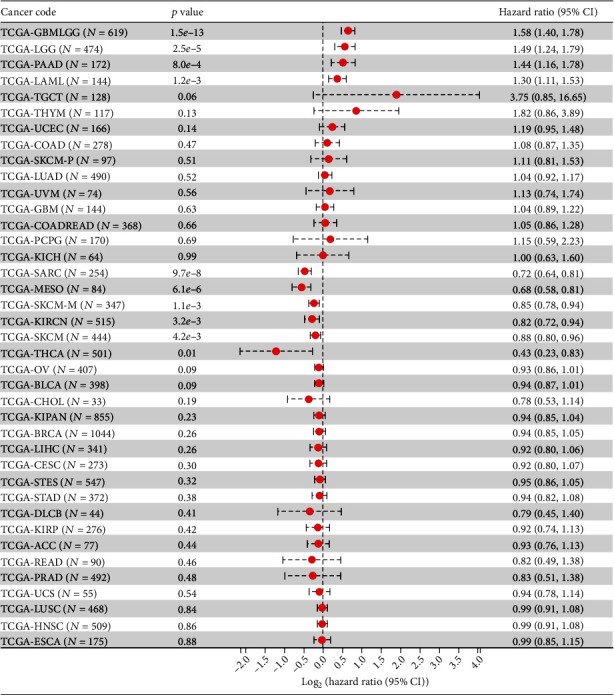
The relationship between RTP4 gene expression and prognosis in 39 types of tumors.

In the analysis process of the relationship between expression level and tumor prognosis, we found that the poor prognosis of 4 tumors was positively correlated with the high expression of RTP4 (GBMLGG (*N* = 619, *p* = 1.5*e* − 13, HR = 1.58 (1.40, 1.78)), LGG (*N* = 474, *p* = 2.5*e* − 5, HR = 1.49 (1.24, 1.79)), LAML (*N* = 144, *p* = 1.2*e* − 3, HR = 1.30 (1.11, 1.53)), and PAAD (*N* = 172, *p* = 8.8*e* − 4, HR = 1.44 (1.16, 1.78))), and the poor prognosis of 6 tumors was positively correlated with the low expression of RTP4 (SARC (*N* = 254, *p* = 9.7*e* − 8, HR = 0.72 (0.64, 0.81)), KIRC (*N* = 515, *p* = 3.2*e* − 3, HR = 0.82 (0.72, 0.94)), THCA (*N* = 501, *p* = 0.01, HR = 0.43 (0.23, 0.83)), MESO (*N* = 84, *p* = 6.1*e* − 6, HR = 0.68 (0.58, 0.81)), SKCM-M (*N* = 347, *p* = 1.1*e* − 3, HR = 0.85 (0.78, 0.94)), and SKCM (*N* = 444, *p* = 4.2*e* − 3, HR = 0.88 (0.80, 0.96))) (Figures 12 and 13).

**Figure 14:**
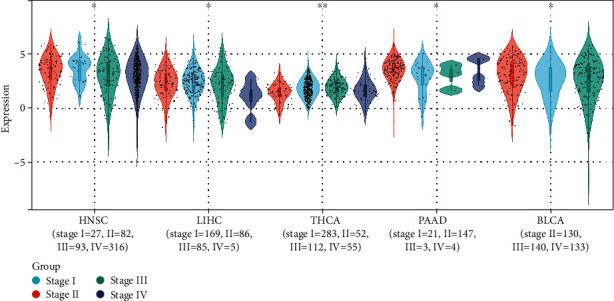
Differences in the expression of RTP4 in different stages in 5 types of tumors. ^∗^0.05, ^∗∗^0.01, ^∗∗∗^0.001, and ^∗∗∗∗^0.0001.

After exploring the expression difference of the RTP4 gene in different clinical stages in each type of tumor, we found significant differences among the 5 types of tumors. (HNSC (stage I = 27, II = 82, III = 93, IV = 316) (*p* = 0.03), LIHC (stage I = 169, II = 86, III = 85, IV = 5) (*p* = 0.04), THCA (stage I = 283, II = 52, III = 112, IV = 55) (*p* = 2.7*e* − 3), PAAD (stage I = 21, II = 147, III = 3, IV = 4) (*p* = 0.02), and BLCA (stage II = 130, III = 140, IV = 133) (*p* = 0.02)) (Figure 14).

## 4. Discussion

MTB can infect many tissues and organs of the human body, causing various tuberculosis-related diseases, such as primary and secondary tuberculosis, tuberculous enteritis, osteoarticular tuberculosis, tuberculous pleurisy, tubercular meningitis, and tuberculous lymphadenitis. Some diseases can be fatal because of delays in diagnosis and treatment.

Tuberculosis is a common infectious disease in developing countries. There are some clinical tests in diagnosing this disease, but those ways have somehow false negatives [14–16]. The histopathologic biopsy is an excellent method to diagnose tuberculosis infection. Due to the difficulty of sampling, some tissues in vivo and patients cannot tolerate the discomfort during the sampling process, so this method is not often used in clinical diagnosis. Sputum culture is considered the gold standard for diagnosing PTB infections. However, as a slow-growing bacteria, clinical doctors may be able to get results in 2-3 months. We conducted this study to reduce the time of diagnosing and improve the diagnostic accuracy of PTB.

Several previous researchers have reported some potential biomarkers for tuberculosis [17, 18]. Our study is aimed at investigating new biomarkers for diagnosing pulmonary tuberculosis related to folate synthesis, and RTP4 was finally identified in training and validated in the validation set.

Folate acid is known as vitamin B9. It is necessary for the body to yield amino acids, RNA, and DNA [19]. It has previously been reported that lacking folic acid may lead to neuropathy and neoplastic diseases [20–22]. Folic acid is also an element for M. tuberculosis. It is believed that folate can affect the synthesis of purines and thymidine by regulating one-carbon transfer reactions as an essential factor, which is vital for bacteria [23, 24]. In recent years, there has been renewed interest in synthesizing antifolic acid drugs due to the increase in clinical drug-resistant tuberculosis cases [25, 26].

The RTP4 is known as a member of the receptor transport protein (RTP), which participates in exporting odorant and taste receptors to the cell surface [27, 28]. Previous studies have suggested that RTP4 helps the GPCR oligomer properly assemble in the endoplasmic reticulum, promotes the dimerization of receptors in the Golgi apparatus, and decreases the ubiquitination of the heterodimers [29]. Other studies have shown that the RTP4 was overexpressed in some connective tissue disorders and parasitic infections [30, 31] and correlated with some cancers [32, 33]. That RTP4 induced by IFN-I could explain this phenomenon and is associated with immune responses.

In our research, we also found several other enriched signaling pathways related to complications of PTB. Previous studies have implicated the NOD-like signaling pathway and Toll-like receptor signaling pathways in idiopathic pulmonary fibrosis [34]. The Hedgehog signaling pathway was reported to be associated with lung cancer. Abnormal activation of this pathway is thought to be related to the development of lung cancer and promotes malignant lung cancer progression with poor prognostic outcomes [35, 36]. These may explain some PTB patients' clinical progression of pulmonary fibrosis and lung cancer. However, in this study, we only found that the folate biosynthesis singling pathways were most associated with the PTB group in training gene sets.

Some previous articles have reported a correlation between the RTP4 gene and certain characteristics of tumors. For example, methylated RTP4 can be considered as a new biomarker for precise diagnosis and treatment of prostate cancer [37] and RTP4 can be considered as an independent indicator to judge the prognosis of oral cancer [38]. RTP4 has also been reported that it is significantly associated with immune cell infiltration and immune checkpoint encoding genes (PDCD1, TIM-3, and LAG3) in melanoma [33].

We worked on seeking the different genes as biomarkers of diagnosing PTB infection through four GEO databases and used a method combing gene set variation analysis with weight gene coexpression network analysis. There were some limitations in this study. Firstly, the validated samples were less than the training samples, and more gene chip samples of patients with TB were validated from the experimental verification. To further explore the relationship between the expression of RTP4 and tumors, we performed a pan-cancer analysis based on TCGA database. It was also found that RTP4 expression was different between some tumor and normal tissues, and it was correlated with the overall survival time and stage of some tumors.

To conclude, our results suggest that RTP4 can be used as a biomarker to identify tuberculosis infection, providing a new perspective on TB diagnosis in clinical.

## Data Availability

Data can be obtained from GEO (https://www.ncbi.nlm.nih.gov/geo/) and TCGA (https://www.cancer.gov/ccg/research/genome-sequencing/tcga).

## Abbreviations

PTB: Pulmonary tuberculosis
HC: Healthy control
WGCNA: Weight gene coexpression network analysis
ROC: Receiver operating characteristic
GSVA: Gene set variation analysis
PPI: Protein-protein interaction network
MTB: Mycobacterium tuberculosis
APC: Antigen-presenting cells
TST: Tuberculin skin test
STRING: Search Tool for the Retrieval of Interacting Genes
AUC: Area under curve
KEGG: Kyoto Encyclopedia of Genes and Genomes
ACC: Adrenocortical carcinoma
BLCA: Bladder urothelial carcinoma
BRCA: Breast invasive carcinoma
CESC: Cervical squamous cell carcinoma and endocervical adenocarcinoma
CHOL: Cholangiocarcinoma
COAD: Colon adenocarcinoma
COADREAD: Colon adenocarcinoma/rectum adenocarcinoma esophageal carcinoma
DLBC: Lymphoid neoplasm diffuse large B-cell lymphoma
ESCA: Esophageal carcinoma
FPPP: FFPE pilot phase II
GBM: Glioblastoma multiforme
GBMLGG: Glioma
HNSC: Head and neck squamous cell carcinoma
KICH: Kidney chromophobe
KIPAN: Pan-kidney cohort (KICH+KIRC+KIRP)
KIRC: Kidney renal clear cell carcinoma
KIRP: Kidney renal papillary cell carcinoma
LAML: Acute myeloid leukemia
LGG: Brain lower grade glioma
LIHC: Liver hepatocellular carcinoma
LUAD: Lung adenocarcinoma
LUSC: Lung squamous cell carcinoma
MESO: Mesothelioma
OV: Ovarian serous cystadenocarcinoma
PAAD: Pancreatic adenocarcinoma
PCPG: Pheochromocytoma and paraganglioma
PRAD: Prostate adenocarcinoma
READ: Rectum adenocarcinoma
SARC: Sarcoma
STAD: Stomach adenocarcinoma
SKCM: Skin cutaneous melanoma
STES: Stomach and esophageal carcinoma
TGCT: Testicular germ cell tumors
THCA: Thyroid carcinoma
THYM: Thymoma
UCEC: Uterine corpus endometrial carcinoma
UCS: Uterine carcinosarcoma
UVM: Uveal melanoma.

## Supplementary Materials

Supplementary 1 Supplementary materials The difference in gene expression between the PTB group and the HC group are shown in supplementary Figure 1, Figure 2, and Figure 3 (supplementary Figure 1: GSE28623, supplementary Figure 2: GSE83456, and supplementary Figure 3: GSE 34608).
