# Supplementary figures and images for: RTP4, a Biomarker Associated with Diagnosing Pulmonary Tuberculosis and Pan-Cancer Analysis
Source: Mediators Inflamm. 2023 Apr 26;2023:2318473. doi: 10.1155/2023/2318473 (PMC10156460; doi:10.1155/2023/2318473)

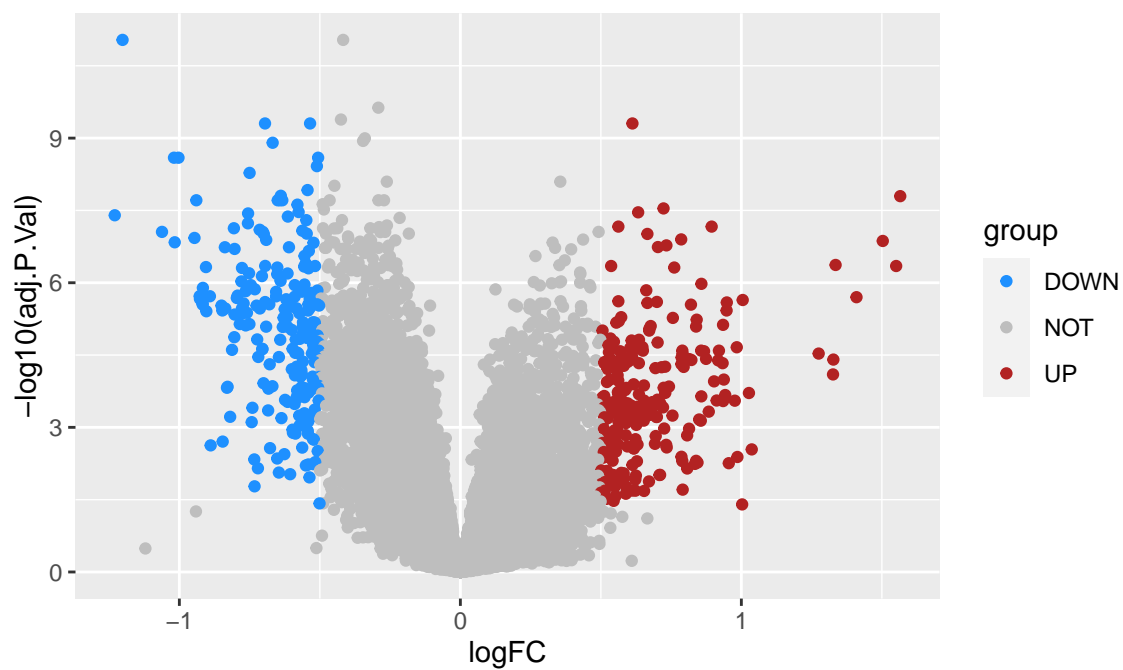

Supplement: Supplementary 1 — Supplementary materials The difference in gene expression between the PTB group and the HC group are shown in supplementary Figure 1, Figure 2, and Figure 3 (supplementary Figure 1: GSE28623, supplementary Figure 2: GSE83456, and supplementary Figure 3: GSE 34608). [file 2318473.f1.zip › Supplement Fig 1.pdf]

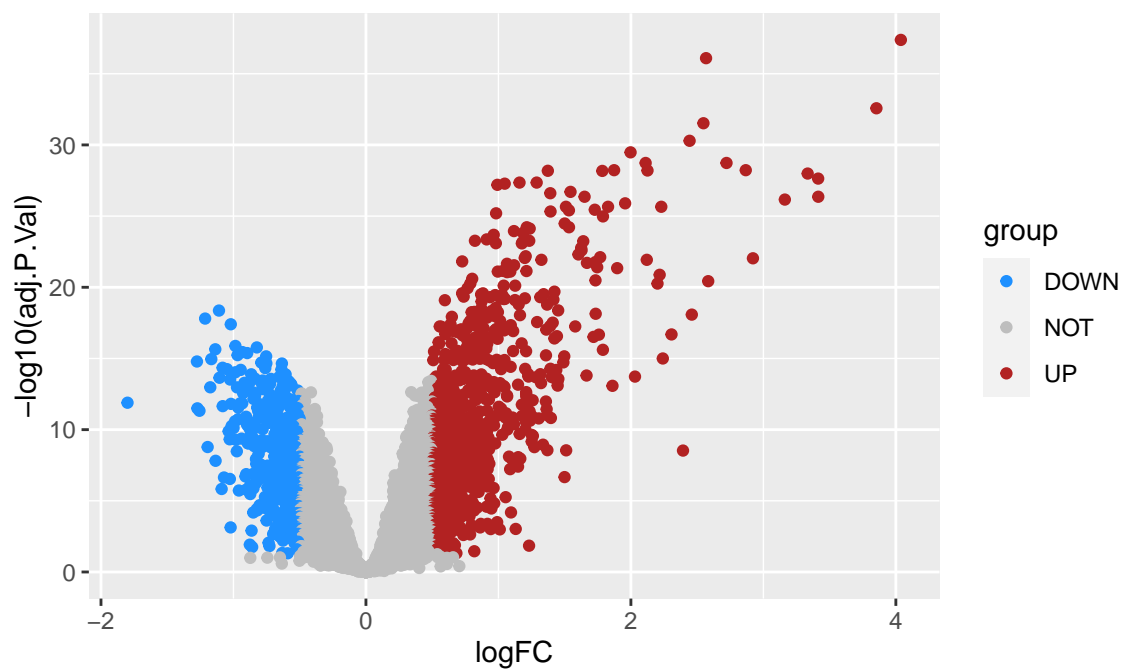

Supplement: Supplementary 1 — Supplementary materials The difference in gene expression between the PTB group and the HC group are shown in supplementary Figure 1, Figure 2, and Figure 3 (supplementary Figure 1: GSE28623, supplementary Figure 2: GSE83456, and supplementary Figure 3: GSE 34608). [file 2318473.f1.zip › Supplement Fig 2 (1).pdf]

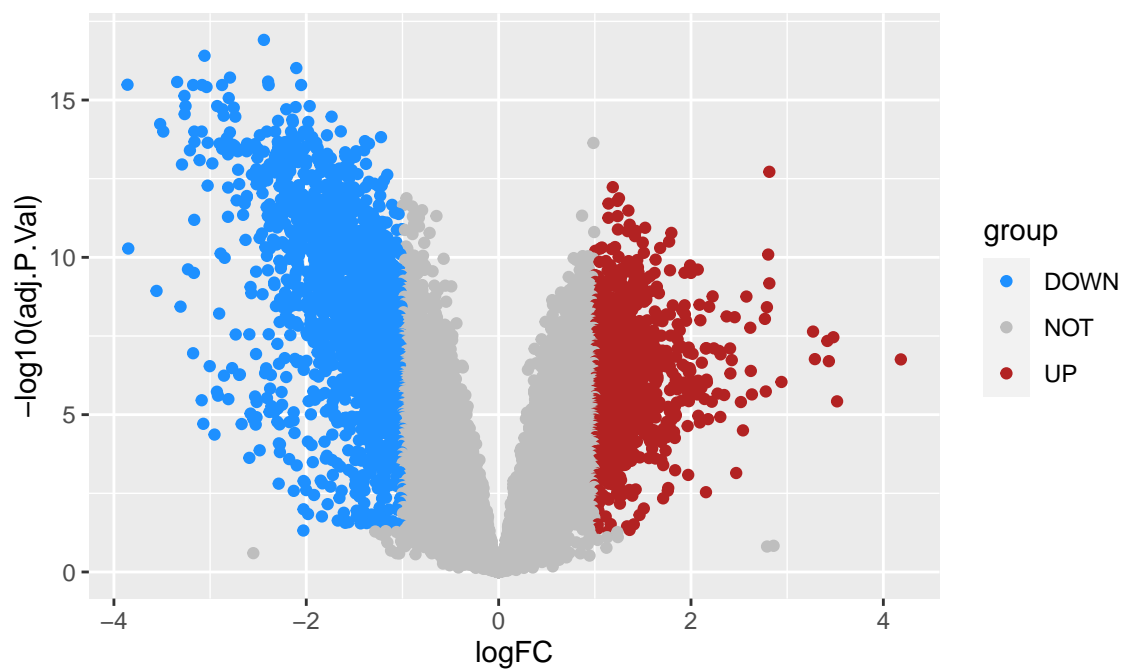

Supplement: Supplementary 1 — Supplementary materials The difference in gene expression between the PTB group and the HC group are shown in supplementary Figure 1, Figure 2, and Figure 3 (supplementary Figure 1: GSE28623, supplementary Figure 2: GSE83456, and supplementary Figure 3: GSE 34608). [file 2318473.f1.zip › Supplement Fig 3.pdf]
